# Perceived importance of walking among hospitalized patients with stroke: a thematic analysis

**DOI:** 10.3389/fneur.2026.1742132

**Published:** 2026-02-19

**Authors:** Shingo Mitsue, Tatsuya Ogawa, Yuji Minamikawa, Shinichi Shimada, Shu Morioka

**Affiliations:** 1 Department of Neurorehabilitation, Graduate School of Health Sciences, Kio University, Nara, Japan; 2 Department of Rehabilitation, Nishiyamato Rehabilitation Hospital, Nara, Japan; 3 Department of Neurosurgery, Itami Kousei Neurosurgical Hospital, Hyogo, Japan; 4 Neuro Rehabilitation Research Center, Kio University, Nara, Japan

**Keywords:** hospital, qualitative research, rehabilitation, stroke, walking

## Abstract

**Introduction:**

Improved walking ability is a common rehabilitation goal for individuals following a stroke. However, the reasons why hospitalized individuals with stroke consider walking to be important are not yet fully understood. This study aimed to elucidate the perceived importance of walking among hospitalized patients with stroke.

**Methods:**

This qualitative study employed thematic analysis. Hospitalized individuals with stroke undergoing gait rehabilitation were purposively sampled to capture variation in sex, age, and walking ability. The participants underwent in-person semi-structured interviews regarding the importance of walking, which were audio-recorded, transcribed verbatim, and systematically coded to generate themes.

**Results:**

A total of 19 patients participated in the study. Thematic analysis revealed six major themes. (1) Resumption of daily life: walking was perceived as essential for returning to pre-stroke activities and routines. (2) Health promotion and prevention of functional decline: participants viewed walking as important for maintaining health and preventing deterioration. (3) Uncomfortable walking: participants described physical and environmental challenges associated with walking. (4) Relationships with others: concerns were expressed about how walking difficulties might affect relationships with family and others. (5) Labeling of decreased walking ability: participants were conscious of how their walking was perceived by others. (6) Social environment: walking was linked to broader social factors such as work and transportation.

**Conclusion:**

The importance of walking for hospitalized patients with stroke ranges from impersonal and generalizable reasons to highly individualized and diverse factors, with implications for individualized walking rehabilitation.

## 1 Introduction

Stroke is a sudden-onset condition, with approximately 80% of people who have experienced stroke experiencing impaired walking ability (1), with gait disturbance as one of the most critical issues (2). Currently, many stroke rehabilitation programs focus on enhancing the biomechanical and neurological aspects of individuals post-stroke (3). While such approaches offer structured methods for functional recovery, the use of standardized assessments and interventions may inadvertently limit the extent to which the preferences and values of people post-stroke are incorporated into their rehabilitation (4). Schoeb and Bürge (5) reported that individuals undergoing physical therapy desire to more actively express their opinions, goals, and preferences. For example, even when a person post-stroke sets “improving walking ability” as a goal, there may be underlying intentions, such as a desire to walk independently, safely, or with a more natural gait. This suggests a potential gap between clinical priorities and patients’ own hopes for recovery (6), indicating the possibility that even among healthcare professionals involved in gait rehabilitation, the perspectives of individuals post-stroke may not be fully captured. Therefore, for post-stroke gait recovery, it is necessary to not only examine the factors that affect walking ability, such as independence, speed, endurance, and quality, but also the relationship between walking performance and the environment (7), and to provide rehabilitation that considers the opinions and preferences of individuals regarding their gait post-stroke.

A report by Bohannon et al. (8) is frequently cited in studies on the importance of and preference for walking among individuals post-stroke. This study found that individuals post-stroke considered walking independence the most critical factor, followed by distance, appearance, and speed. In addition, Combs et al. (9) reported that individuals with chronic stroke tend to prioritize walking distance over walking speed during outdoor activities. Furthermore, a qualitative analysis of the reasons for preference for specific walking elements revealed that, in terms of walking distance, some participants desired engagement in community activities and social participation, whereas others wished to walk longer distances, even at a slower speed. Some participants prioritized walking speed because they wanted to reach their destination more quickly. These findings suggest that the perceived importance of walking among people post-stroke may vary depending on background factors, such as the lifestyle roles they wish to resume and the range of their desired activity. Although Bohannon et al. (8) identified which walking elements individuals post-stroke prioritize, the reasons behind these choices were not the main focus of the study. Similarly, although Combs et al. (9) highlighted walking distance and speed, the motivations for prioritizing other walking factors remain open for further investigation. Building on these valuable insights, additional research is needed to better understand why individuals consider walking important after stroke and how their individual backgrounds affect these preferences.

The subacute phase, commonly defined as the period from approximately 7 days to 6 months after stroke onset (10), is the primary period during which hospitalized people with stroke receive intensive rehabilitation. During this phase, the incorporation of walking training is recommended in clinical practice guidelines (11), and the assessment of multiple walking-related outcomes is also considered necessary (12). During this period, it is essential to support individuals in achieving the highest possible functional level through interventions focused on improving physical function, sensorimotor impairments, and activities of daily living (13, 14). In addition, individuals post-stroke seek to reconstruct their identities and roles in preparation for reintegration into society after hospital discharge (6). Thus, the period of inpatient rehabilitation for people post-stroke is positioned as a critical phase not only for focusing on physical recovery but also for exploring post-discharge life and reconstructing a way of life, considering the individual’s social context. Based on these findings, in addition to interventions aimed at improving the walking ability, approaches that incorporate the psychosocial aspects (15) of individuals post-stroke are required for gait rehabilitation. However, knowledge of such approaches is limited. Exploring the perceptions of walking among people post-stroke during this stage of rehabilitation could help improve the understanding of the broader meaning of walking ability, which may produce rehabilitation interventions that align with individual preferences and their perceived importance.

The importance of walking varies among stroke survivors, making qualitative methods well suited to exploring their values and experiences. Therefore, this study aimed to clarify why hospitalized individuals post-stroke perceive walking to be important during inpatient rehabilitation.

## 2 Materials and methods

This study used a qualitative design with semi-structured interviews and thematic analysis, which served as the methodological orientation, as described by Braun and Clarke (16), to explore the reasons hospitalized people with stroke consider walking important. This study was conducted according to the Consolidated Criteria for Reporting Qualitative Research (COREQ) checklist (17) (Supplementary file 1).

### 2.1 Participants

Participants were recruited from the convalescent rehabilitation ward of Itami Kosei Neurosurgical Hospital in Hyogo, Japan. They were undergoing gait rehabilitation following a confirmed medical diagnosis of stroke, specifically intracerebral hemorrhage or cerebral infarction. To capture diverse experiences and perspectives on walking, individuals who met the inclusion criteria were purposively sampled as potential participants. The selection considered the following criteria: (A) variation in walking ability, with a Functional Ambulation Categories (FAC) score of 1–5 (18); (B) balanced representation of both sexes; and (C) inclusion of middle-aged to older adults (aged ≥40 years). Participants were excluded if medical records by the attending physician indicated evident cognitive impairment or aphasia that could interfere with their ability to participate in the interview or if chart information showed that they were unable to walk prior to hospitalization. Eligible individuals were identified by the first author (SMi), who initially provided a brief overview of the study. Those who expressed interest were informed that participation would involve an interview focusing on walking during hospitalization and sharing their personal experiences related to walking. They were then given a detailed explanation of the study’s purpose, procedures, and ethical considerations, and individuals who expressed willingness to discuss walking and provided written informed consent were subsequently included. Data were collected between June and October 2023.

### 2.2 Data collection

All clinical assessments were conducted by the attending physiotherapists within a 7-day period, during which semi-structured interviews were conducted separately by the first author (SMi), a male physical therapist with clinical experience in stroke rehabilitation.

The demographic characteristics of the participants, including age, sex, and the time since stroke onset, were obtained from the participants’ medical records. Stroke-related characteristics were assessed using the National Institutes of Health Stroke Scale (NIHSS) for neurological severity (19, 20), the Fugl-Meyer Assessment-Lower Extremity for lower-limb motor impairment (21), and the Berg Balance Scale for balance evaluation (22). Walking ability was assessed through multiple measures: FAC for walking independence, 10-m walk test for comfortable walking speed (23, 24), 6-min walk test for walking endurance (25).

The interviews were conducted in Japanese and audio-recorded (AutoMemo S, SOURCENEXT) in a private room that ensured confidentiality and provided a comfortable conversational environment. The interviews were conducted in a one-on-one face-to-face format between the interviewer and participant. The interviewer was affiliated with the research institution and was not the participants’ primary physical therapist. Although the interviewer had occasionally provided physical therapy as a covering therapist during the absence of the primary therapist, they were not involved in the participants’ ongoing care at the time of the interview. Before the semi-structured interviews, the Community Integration Questionnaire (CIQ) (26) was administered to investigate participants’ history, including pre-stroke activities and social participation. Understanding the history of each participant helped facilitate more focused and meaningful dialogue during the interview.

### 2.3 Questionnaires and semi-structured interviews

Based on a study by Combs et al. (9), the discussion of the importance of walking focused on identifying the walking elements that the participants recognized as important. Specifically, referring to a previous study by Bohannon et al. (8), which examined walking preferences based on four elements—independence, distance, appearance, and speed—this study additionally incorporated balance, as walking can be evaluated from a safety perspective (27). Based on these previous studies, five walking elements—independence, distance, appearance, speed, and balance—were adopted as the theoretical framework for the present study. In accordance with this framework, a questionnaire was developed to enable the elicitation of the relative importance of each walking element from the participant’s perspective, as demonstrated by Bohannon et al. (8). This questionnaire was used to assess which walking elements were prioritized by the participants. The operational definitions of these five walking elements were based on previous studies (8, 28): independence was defined as “the ability to walk without assistance or supervision,” distance as “the ability to walk longer distances,” balance as “the ability to walk safely,” appearance as “how normal you look when you walk,” and speed as “the ability to walk faster.” After explaining these definitions, the participants were asked to rank the five elements in the order of priority. Subsequently, the interviewer conducted a semi-structured interview focusing only on the single elements that the participant ranked as the most important.

Semi-structured interviews were conducted based on the methods described by Combs et al. (9). To begin the interview, an introductory question was posed in a “Why” format: “Why do you consider the element you ranked first to be the most important to you at present?” However, it has been noted that “why” questions can be abstract and may be difficult for some participants to answer (29). Therefore, in the present study, the 5W1H framework (30) was utilized as a set of follow-up questions to further explore the context and background underlying the participant’s prioritization of a particular walking element. Accordingly, following the initial question, specific and context-dependent follow-up questions were asked using the 5W1H framework (Who, Where, When, What, How) to improve the understanding of the perceived importance of walking. Specifically, the following questions were posed: Who – “Who considers the element you ranked first to be important?” Where – “In what situations do you consider the first-ranked element to be important?” When – “When do you consider the first-ranked element to be important?’ What – “What do you consider the first-ranked element to be important for?” and How – “How do you perceive the first-ranked element?” After completing the interview, the main points of the conversation were reviewed and the participants were asked to confirm whether their ranking of the most important elements remained unchanged.

### 2.4 Data analysis

Thematic analysis was performed using the procedure described by Braun and Clarke (16), with data analyzed using Nvivo14 (LUMIVERO). The analysis followed an inductive approach. Two authors (SMi and TO), with backgrounds in physical therapy and extensive experience in gait rehabilitation for individuals with stroke, were responsible for the qualitative analysis. They are affiliated with different institutions and have clinical experience working with patients in the acute, subacute, and chronic phases of stroke recovery. SMi has 5 years of clinical experience; TO has 16 years of clinical experience and has received training in qualitative research methodologies, including practical experience in qualitative analysis. In addition, the research team included professionals from multiple disciplines: an occupational therapist (YM), a physician (SS), and a physical therapist and university professor (SMo).

First, interview data were transcribed verbatim to create a detailed transcript. Second, the transcripts were read repeatedly to enhance their understanding, and coding was performed to extract data related to the importance of walking. Thereafter, the extracted data were organized into initial codes. Third, these initial codes, which represent common meanings related to the importance of walking, were grouped into provisional categories. Fourth, similar provisional categories were combined to form themes. Finally, the focus of each theme and sub-theme was clarified based on the perspectives of the participants, who were named with clearly defined meanings. To ensure the reliability and credibility of the qualitative analysis, investigator triangulation (31) was employed. Two authors (SMi and TO), both with clinical and research expertise in stroke rehabilitation, independently conducted the analysis up to the third stage: repeated reading of the transcripts, initial coding, and grouping into provisional categories. Subsequently, all coding results were shared and discussed between the two authors (SMi and TO). In cases of discrepancy, discussions continued until a consensus was reached, leading to the final determination of subthemes and theme names. In addition, third team members with different disciplinary backgrounds (YM and SMo) reviewed the categories and themes to enhance the validity of the data analysis regarding the importance of walking. Data collection and data analysis were conducted concurrently. Subsequently, participants were enrolled in stages as the analysis progressed. After each stage, we evaluated whether new themes or insights emerged. Data saturation was reached when no new insights emerged, existing data began to repeat, and further data collection was considered redundant (32).

## 3 Results

Twenty hospitalized people with stroke were initially selected through purposive sampling. However, one participant expressed that walking was not important to them and clearly stated, “I do not want to talk about it.” Based on this response, it was judged that continuing the interview would be inappropriate. Therefore, the interview was terminated early, and the participant’s data were excluded from the analysis. As a result, the final study sample consisted of 19 participants (Table 1). The participants were predominantly older adults, with a median age of 72 years (interquartile range [IQR], 69.5–74.5), and included 11 males. The median time since stroke onset was 37 days (IQR, 35.5–40), indicating that most participants were undergoing inpatient rehabilitation. Most participants were able to walk with supervision or minimal assistance, as reflected by a median Functional Ambulation Categories (FAC) score of 4 (IQR, 3–4). The median Community Integration Questionnaire (CIQ) score was 13 (IQR, 9–18), indicating variation in participants’ levels of community integration prior to hospitalization. Regarding the walking element considered most important by the participants, nine prioritized balance, seven prioritized independence, two prioritized distance, and one prioritized appearance.

**Table 1:** Demographic, stroke characteristics (*n* = 19).

Characteristic	Median (IQR)	Range	*n* (%)
Age (years)	72 (69.5–74.5)	48–82	–
Sex			
Male	–	–	11 (57.9)
Female	–	–	8 (42.1)
Time since stroke (days)	37 (35.5–40)	31–210	–
NIHSS	3 (2–5)	0–11	–
FMA-LE	32 (28.5–34)	8–34	–
BBS	52 (44.5–54)	4–56	–
FAC	4 (3–4)	1–5	–
10MWT (m/s)	0.9 (0.5–1.2)	0–1.5	–
6MWT (m)	394 (214–423.5)	0–553	–
CIQ	13 (9–18)	2–26	–
The most important			
Independence	–		7 (36.8)
Distance	–		2 (10.5)
Appearance	–		1 (5.3)
Speed	–		0 (0)
Balance	–		9 (47.4)

IQR, inter quartile range; NIHSS, National Institutes of Health Stroke Scale; FMA-LE, Fugl-Meyer Assessment-Lower Extremity; BBS, Berg Balance Scale; FAC, Functional Ambulation Categories; 10MWT, 10-m walk test (comfortable); 6MWT, 6-min walk test; CIQ, Community Integration Questionnaire.

The average interview duration was 11 min and 43 s (range: 8 min and 8 s to 16 min and 56 s). Although the average interview duration was relatively short, this reflected the focused approach adopted in the study. In the same session, immediately prior to the semi-structured interview, data were collected using the CIQ and a prioritization questionnaire on walking elements. This process allowed the interviews on the importance of walking to remain concise while allowing participants to reflect and elaborate on the aspects they found most significant.

Thematic analysis of the importance of walking identified six key themes (Table 2). The following themes illustrate how stroke survivors undergoing inpatient rehabilitation perceive the importance of walking in relation to their prior daily lives, exercise habits, relationships with others, and broader social and institutional contexts.

**Table 2:** Main themes and subthemes of the importance of walking.

Main theme	Subthemes
Resumption of daily life	Activities and participation
	Autonomous life
Health promotion and prevention of functional decline	Walking as exercise
	Exercise in familiar community or care settings
	Concern about becoming bedridden
Uncomfortable walking	Stumbling indoors
	Anxiety about outdoor environment
	Feeling unsteady
	Visual disturbances
Relationships with others	Avoiding burdening others
	Consideration for family members
	Maintaining established family roles
Labeling of decreased walking ability	Discomfort with assistance
	Remarks from others
	Concern about others’ perceptions
Social environment	Institutional Issues
	Mobility-related resource issues
	Economic Issues

### 3.1 Theme 1: resumption of daily life

Walking is a fundamental aspect of leading an independent life, and it is considered an essential starting point for maintaining previous lifestyle habits and daily activities.

“Walking is everything. If I cannot walk on my own, nothing will start. I would not be able to get out of bed in the morning. Without this, there was no beginning.” (ID 01, The most important: Independence)

Many participants considered walking a means of regaining mobility to resume the roles, activities, and social participation that they valued before experiencing a stroke. Examples include basic activities necessary for daily living such as going to the bathroom; instrumental activities such as shopping; and leisure activities such as hobbies.

“I need to go shopping. You know about the supermarket. I have to walk there to shop.” (ID 01, The most important: Independence)

“When I get home, I need to be able to go to the bathroom by myself. In the hospital, there is always someone around. However, at home, there is sometimes no one there. Like when my wife goes out, shopping. At those times, I had to go to the bathroom on my own. I really need to be able to do so, at least that much.” (ID 07, The most important: Independence)

“I also practice a traditional art that requires carrying items with both hands, so that's another reason. There's even a specific way to step when turning. Everything follows a set pattern. it's something that naturally becomes part of you. So when I return home, I’d like to try it once to see if it still comes naturally to me. I wonder about that.” (ID 03, The most important: Balance)

“I think it would be fun to go anywhere freely. I used to enjoy walking freely through public spaces. I want to experience that again.” (ID 08, The most important: Distance)

Additionally, one participant perceived walking to be very natural and questioned the premise of being asked about its importance. This perspective contrasted with the predominant narratives that framed walking as a goal to be regained, highlighting a taken-for-granted understanding of walking as an unquestioned part of everyday life.

“This is a pointless question. It is like asking, "Why do you eat?" Well, to stay alive, right? Same thing.” (ID 04, The most important: Independence)

### 3.2 Theme 2: health promotion and prevention of functional decline

Walking is recognized as an essential activity, as the act of walking itself serves as an exercise that contributes to the maintenance of physical function and overall health. The participants emphasized the necessity of walking to sustain their habits of taking strolls in familiar neighborhoods or engaging in physical activities at rehabilitation facilities after discharge. At the same time, some participants acknowledged ambivalence, recognizing that avoiding activity would be easier but ultimately undesirable.

“Even if I just want to take a stroll, you know? I would like to go out for a morning walk slightly earlier for training. Kind of like a rehab, you know?” (ID 04, The most important: Independence)

“Honestly, not going at all is the easiest, but that is not how it works. Gotta get some exercise. There’s a nearby place where people exercise, mostly older adults.” (ID 02, The most important: Balance)

Furthermore, some participants associated walking with the prevention of becoming bedridden, perceiving it as a crucial factor in maintaining independence and quality of life.

“Until now, I have been able to walk a little, so it's okay. But if I collapse again next time, I feel like that will be it… I will probably end up being bedridden.” (ID 12, The most important: Balance)

### 3.3 Theme 3: uncomfortable walking

Many participants recognized the effects of post-stroke sequelae and physical and environmental barriers during walking. Consequently, they experienced fear and anxiety about walking indoors and outdoors, which caused them to prioritize walking as a way to overcome these challenges. The primary post-stroke impairments reported included foot drop, oculomotor nerve palsy, and visual disturbances, such as diplopia.

“Even on a flat surface, I still trip. I guess it's the material that's making me catch my foot, or maybe I am just not lifting it high enough… It feels like I almost stumble a bit like my foot is not coming up properly and getting caught.” (ID 03, The most important: Balance)

“Right now, I am fine looking straight ahead or to the left. Even when turning right is okay, but when I look over my right shoulder, my eyes do not keep up, and I feel a bit unsteady.” (ID 19, The most important: Balance)

Regarding the physical and environmental barriers affecting outdoor walking, the participants identified hazards such as bicycles, crowded areas, uneven surfaces (curbs and steps), and crosswalks at traffic signals as major concerns.

“Like when someone is coming towards me, I have to step aside. If someone pushes me, I may fall. When it is crowded, I wonder if I can stay in the middle without being jostled. People push from behind, from the front… And if a bike suddenly comes at me from ahead, it is dangerous.” (ID 10, The most important: Balance)

“When walking outside, steps and uneven surfaces are always present. Even in the park, if there's a little hill, my foot sometimes just sinks down awkwardly there.” (ID 17, The most important: Balance)

“What worries me is the traffic lights. What if I cannot make it across time?” (ID 18, The most important: Balance)

In addition, some participants expressed concerns about their ability to walk independently outdoors after discharge, as they received support from others during practice sessions when hospitalized.

“Right now, I always have someone next to me, so I do not feel worried. But when I have to go out alone… I wonder if I will be able to go shopping by myself.” (ID 05, The most important: Balance)

### 3.4 Theme 4: relationships with others

The importance of walking was not limited to personal concerns but also evoked social emotions related to consideration for others and the potential burden one might place on them. In other words, walking had significance beyond physical function or individual autonomy, taking on deeper meaning within the context of relationships with others. Moreover, the narratives emphasized the emotional distress not necessarily related to the actual ability to walk independently but rather to the concern that walking might impose a burden on others.

“Walking is something you are supposed to be able to do on your own, right? It's a personal thing. However, when that changes, you end up causing trouble to others. You become a burden. You put extra pressure on people around you. That is the part that really gets to me.” (ID 10, The most important: Balance)

Another important feature of this theme was the participants’ concern for their families, particularly spouses and children. The participants strongly expressed a desire not to place a burden or cause inconvenience to their families in relation to walking and voiced concerns about the possibility that the balance of their previously established family relationships might change. Underlying these concerns was a deep-seated wish to remain an equal and mutually supportive partner to their spouse, as well as to maintain their role as an independent parent towards their children.

“I just want to walk on my own as much as possible without causing trouble to anyone. Most importantly, I wanted my husband to feel at ease.” (ID 13, The most important: Independence)

“If my legs don't work, I'll become a burden to my child.” (ID 14, The most important: Balance)

### 3.5 Theme 5: labeling of decreased walking ability

The importance of walking extends beyond personal concerns and affects self-perception through the perspective of others, as well as psychological aspects related to the perception of disability. For example, some participants expressed discomfort when their walking style was pointed out or when others anticipated assisting them.

“If I walk more naturally, no one's gonna say, 'Hey, you are leaning to one side.' I just do not like people pointing it out, that's all … It's hard to walk when someone is too close. We both have to watch our steps, you know?” (ID 06, The most important: Balance)

In addition, among the patients who were conscious of their gait from an external perspective, some were concerned about how their gait would appear while walking before a large crowd. Others expressed a desire to avoid being perceived as a stroke patient based on their gait.

“People can tell, right? If you are leaning or wobbling. they will think, 'Oh, I guess they still have aftereffects from stroke.” (ID 06, The most important: Balance)

“When you’re involved in certain cultural activities, walking steadily and rhythmically becomes essential. Performing in front of others adds extra pressure.” (ID 03, The most important: Balance)

“Yeah, you can totally tell just by someone's walk—how bad their condition is. I just want to make this as unnoticeable as possible.” (ID 09, The most important: Appearance)

### 3.6 Theme 6: social environment

The importance of walking was described not merely as a means to return to work but as a practical mode of mobility for engaging in labor and regaining income, thereby maintaining the financial stability of the household. In other words, walking in this context was positioned not only as a way to resume pre-stroke activities and participation but also as a social resource that enables economic stability in daily life.

“If I had the money, I’d retire. But I’ve gotta keep working — I just can’t afford to be stuck in bed.” (ID 07, The most important: Independence)

Another participant highlighted the importance of walking by comparing it with the cost of institutional care and expressed a strong desire to continue living at home. Because institutionalization was perceived as unrealistic due to financial reasons, there was a clear recognition that being able to walk was necessary to continue living at home.

“I heard those facilities are expensive. It seems difficult without sufficient financial support.” (ID 01, The most important: Independence)

In addition, some patients reported that their families advised them to stop driving after hospitalization, which led them to re-evaluate walking as an alternative means of mobility. Driving had previously served as a central means of supporting daily life, and its restriction prompted a renewed recognition of the importance of walking.

“My family encouraged me to stop driving because of the risk. That made me think more seriously about walking.” (ID 12, The most important: Balance).

One participant acknowledged that losing the ability to walk would disrupt their daily life. The participant who made this statement had previously cared for a family member at home as the primary caregiver. Based on this personal experience, they recognized the limitations of current support systems. This background further reinforced their sense of the importance of being able to walk.

“Although support systems and care plans are said to be in place, I often felt that they didn’t fully meet people’s real needs. There always seemed to be gaps in the care provided.” (ID 01, The most important: Independence)

## 4 Discussion

This study aimed to provide a better understanding of why hospitalized individuals post-stroke in rehabilitation wards prioritize walking. The study findings suggest that the significance of walking among this population ranged from generalized non-personal reasons to highly individualized and diverse motivations. A novel contribution of this study is the identification of this wide spectrum of meaning attributed to walking, which had not been fully explored in previous qualitative research on walking among individuals post-stroke. Furthermore, this study emphasizes the importance of considering the perspectives, opinions, and preferences of individuals post-stroke regarding walking during gait rehabilitation.

Patients undergoing rehabilitation after stroke considered walking essential for resuming their activities and independent living before stroke onset. They evaluated their current physical condition to assess the feasibility (33) of walking after discharge. Walking was perceived as crucial in terms of mobility (34), enabling participants to move from one place to another for basic daily activities such as using the toilet, instrumental activities such as shopping, or leisure activities such as visiting familiar locations in their community. Besides, uncertainty (15) regarding whether they could return to their previous way of life was evident from their perception of walking. Furthermore, because subacute stroke survivors are in a transitional phase from a biomedical model to a psychosocial model (35), their focus on recovery may shift from the level of bodily functions and structures to aspects of daily life that they value (36, 37). Although individuals post-stroke are not thought to have a clear understanding of these models, their narratives revealed an attitude of engaging with walking-related challenges and envisioning their future lives by reflecting on their current walking abilities in relation to their life history. This tendency can be regarded as a characteristic feature within the context of inpatient rehabilitation. However, Notkin et al. (38) pointed out that when stroke survivors transition from hospital to community rehabilitation, the rehabilitation goals and plans do not align with the patients’ needs. Therefore, it is important to encourage patient involvement in the goal-setting process and develop walking rehabilitation plans that reflect their life history.

Although walking was recognized as an essential form of exercise for maintaining health after discharge, the participants expressed concerns and anxieties regarding outdoor environments, emphasizing the importance of overcoming these fears. This is consistent with the qualitative meta-synthesis by Li et al. (39), which identifies environmental factors and fear of falling as barriers to physical activity for people post-stroke, highlighting the importance of overcoming these fears and providing support to promote physical activity. Engaging in familiar activities, such as walking (40), rather than structured exercise programs, may be easier to integrate into daily routines. Walking is a widely accessible exercise that supports the maintenance of physical (41) and cognitive (42) functions because it does not require specific equipment and can be performed anywhere (43). The findings of this study indicate that the participants placed importance on walking as a form of exercise in their post-discharge living environments. However, many participants reported experiencing physical and environmental barriers when walking indoors and outdoors, leading to walking-related fear and anxiety. Stroke survivors often exhibit reduced adaptability when adjusting their gait according to environmental conditions (44). Moreover, qualitative research on outdoor walking has highlighted the difficulties experienced by stroke survivors when transitioning from controlled hospital environments to various physical environments (45), such as roads in their homes or communities. This discrepancy is associated with the negative emotional responses related to outdoor mobility. These negative emotions may stem from heightened vigilance. Individuals post-stroke desire relief from concerns about their surroundings and external factors when walking outdoors (46, 47), indicative of their exploration of strategies to adapt to different environments (47). As familiarity with one’s living environment plays a role in promoting post-stroke participation (48), it is necessary to collect information about participants’ history, living environments, and urban layouts during hospitalization (49). Implementing walking training that aligns as much as possible with the patient’s original home environment and supporting the development of adaptation strategies is essential for facilitating reintegration.

Although individuals post-stroke expressed concerns about potential changes in their relationships with others, including spouses, owing to their walking ability, they also placed importance on avoiding impaired gait. Additionally, although caregiver availability (home support) is one of the strongest predictors of discharge (50), stroke survivors often experience feelings of not wanting to burden their spouses or children with care responsibilities and fear that they might become a burden (51). In addition, the loss of family and social roles after discharge leads to a state of dependence, ultimately resulting in a loss of self-esteem (15, 52). A sense of belonging and connection in interpersonal relationships is believed to be related to an individual’s perception of contributing to these relationships (53). In line with this, a systematic review by Lo et al. (54) reported that the physical changes caused by stroke affects an individual’s relationships with others. Although social relationships provide support and encouragement, stroke survivors often worry that a decline in walking ability may alter or disrupt these relationships, leading to psychological distress. In addition, participants were highly conscious of how others perceived them while walking. This aligns with previous studies on upper limb impairment after stroke (55), which suggests that societal perceptions of physical disability often carry negative emotions and stigmas (56, 57), making individuals post-stroke feel shame for standing out as disabled and overly concerned about others’ reactions (58). Such concerns may also increase psychological stress and excessive self-monitoring during walking, which could potentially lead to negative effects on motor performance (59). Therefore, supporting stroke recovery requires the consideration of not only physical function but also psychosocial factors (60). The same perspective should be applied to walking rehabilitation during hospitalization, emphasizing the psychological and social aspects of physical improvement. For example, goal setting is effective in improving health-related quality of life (61) and anxiety (62) and incorporating it into gait rehabilitation may be beneficial.

The inevitable changes in employment and threats to daily life caused by walking difficulties have extended to broader social policy issues. Mobility-related challenges are affected by various resources available to stroke survivors, including financial aspects such as income, pre-stroke working conditions, and social support (45). However, the return-to-work rate for chronic stroke survivors remains notably low (63), and walking ability is the only factor that is significantly associated with job performance (64). In addition, employment is closely associated with self-esteem, and some individuals associate maintaining occupational roles with a sense of identity (51). Given that stroke survivors tend to have a long-term desire to return to work and resume their roles (65), rehabilitation programs should be designed to maximize their participation by considering individual working conditions and environments from the early stages of hospitalization.

Furthermore, individuals with lower ability to perform activities of daily living are more likely to be discharged to external care facilities instead of returning home (66). Generally, the cost of nursing facilities is significantly higher than that of home-based care (67). Furthermore, to utilize medical services upon discharge, individuals must recognize the services available to them (68). However, some healthcare services may not be properly recognized, creating a barrier to access optional services (69). Consequently, walking ability issues may be related to determining the functional discharge destinations for hospitalized stroke survivors. In cases where individuals cannot resolve these challenges independently, the focus may shift towards broader societal perspectives and support systems.

This study highlights the importance of actively involving stroke survivors in rehabilitation planning and developing walking adaptation strategies based on their life history and community environment. Additionally, incorporating goals that consider interpersonal relationships is crucial for supporting the psychosocial aspects of walking after a stroke. Furthermore, walking ability issues significantly impact discharge planning, making it necessary to provide information about healthcare-related social systems tailored to the individual’s circumstances. Future research should explore how individuals post-stroke experience and interpret walking over time. In particular, longitudinal qualitative studies are essential to elucidate how perceptions of walking and psychosocial factors evolve throughout the recovery process.

### 4.1 Limitations

The findings of this study provide new insights into the importance of walking among hospitalized stroke survivors. However, this study had some limitations that must be acknowledged. Although this study aimed to sample participants with a balanced range of stroke severities, the challenges in conducting interviews and obtaining consent resulted in a group of participants primarily composed of mild cases in the early subacute phase. In addition, all participants were sampled from a single rehabilitation ward, which may have affected the results because of the specific rehabilitation interventions provided at that facility. Furthermore, because the study was conducted in Japan, generalizability may be limited by the sociocultural factors unique to this setting. Finally, the relatively short duration of the interviews may have limited the depth or breadth of participants’ narratives, and longer interviews might have yielded additional or more diverse perspectives.

## 5 Conclusion

Walking is important to stroke survivors during hospitalization, with reason ranging from generalized and non-personal reasons to highly individualized and diverse motivations. Walking is essential for resuming pre-stroke daily life and maintaining health; however, concerns about walking indoors and outdoors highlight the need for rehabilitation that aligns with the living environments of individuals post-stroke. In addition, walking is affected by interpersonal relationships, as patients often experience a desire not to burden others while striving to maintain their self-esteem and identity, underscoring the need for individualized consideration. Therefore, incorporating rehabilitation goals that address not only physical capacity but also interpersonal and psychosocial concerns is essential. Furthermore, inevitable changes in employment and threats to daily life caused by walking difficulties have been connected to broader social policy issues. From these perspectives, it is crucial for healthcare professionals to understand the personal significance that stroke survivors attribute to walking when designing post-stroke gait rehabilitation programs.

## Data Availability

The raw data supporting the conclusions of this article will be made available by the authors, without undue reservation.
